# TIM3 and TIGIT-expressing CD4 T cells are impacted by kidney transplantation and associated with risk of infection

**DOI:** 10.3389/fimmu.2025.1550154

**Published:** 2025-05-22

**Authors:** Harry Pickering, Subha Sen, Monica Cappelletti, Erik L. Lum, Suphamai Bunnapradist, Elaine F. Reed, Joanna M. Schaenman

**Affiliations:** ^1^ Department of Pathology and Laboratory Medicine, David Geffen School of Medicine, University of California Los Angeles, Los Angeles, CA, United States; ^2^ Division of Nephrology, David Geffen School of Medicine, University of California Los Angeles, Los Angeles, CA, United States; ^3^ Division of Infectious Diseases, Department of Medicine, David Geffen School of Medicine, University of California Los Angeles, Los Angeles, CA, United States

**Keywords:** T cell, immunosuppression, antithymocyte globulin, kidney transplantation, infection

## Abstract

**Introduction:**

Older kidney transplant patients experience higher rates of infection compared with younger transplant patients suggesting the impact of age-associated immune dysfunction. However, little is known about the impact of immunosuppression including antithymocyte globulin (ATG) induction, as well as whether T cell subtypes can predict risk for infection.

**Methods:**

We collected blood from 91 patients before and then 3 months after kidney transplantation and analyzed CD4 and CD8 T cell phenotypes to determine the impact of immunosuppression on immune maturation, senescence, and infection.

**Results:**

After transplantation the number of naïve T cells decreased overall, while TIM3-expressing naïve and central memory (CM) CD4 T cell frequency increased, with more striking change in patients receiving ATG compared with basiliximab induction. Transplantation also led to increased frequency of TIGIT-expressing effector memory (EM) CD4 T cells and senescent TIGIT and KLRG1-expressing CD8 T cells. Decreased frequencies of naïve CD4 and CD8 T cells (p=0.016 and p=0.038, respectively) and increased frequency of CD4 CM and EM TIGIT+ T cells (p=0.022) were associated with development of infection. A model incorporating increased frequency CD4 EM TIGIT+ T cells and ATG induction was predictive of development of infection after kidney transplantation (HR 3.73, CI 1.08-12.9).

**Discussion:**

Increased frequency of TIM3 and TIGIT markers associated with T cell experience and senescence was a notable phenotypic change associated with transplantation and induction and maintenance immunosuppression. Incorporation of TIGIT expression and induction type into an infection prediction model holds promise for risk stratification and individualization of immunosuppression to decrease risk of adverse outcomes, especially for older patients.

## 1 Introduction

Although the field of kidney transplantation continues to make significant strides forward in patient and allograft survival (1), transplant recipients continue to suffer significant morbidity and mortality from infection, especially for the growing numbers of older patients (2). Despite differing levels of immune function present prior to transplantation, we continue to use a one-size-fits all approach for immunosuppression. This is despite the fact that increased patient age is known to be associated with immune senescence and impaired control of infection (3). There continues to be a knowledge gap in how transplantation impacts the immune system as well as which immune cell phenotypes are most associated with infection.

CD4 and CD8 T cells are the immune cell type most critical for avoidance of opportunistic infection (4). T cell function is especially important for control of viral infections, with increased risk associated with older patient age and immune dysfunction (5–7). Some authors have looked at levels of T cells at a relatively basic level in terms of CD4+ and CD8+ T cell percentages after transplantation (8, 9). However, there remains a lack of information regarding specific subtypes associated with infection and aging such as T cell immunoglobulin and ITIM domain (TIGIT) and Killer cell lectin-like receptor subfamily G member 1 (KLRG1) as well as T regulatory cells (10–12). The co-inhibitory receptor TIM-3 is also notable for its role in NK and T cell dysfunction (10).

Our previous work focused on post-transplant immune phenotype and association between T cell maturation and senescence in terms of infection risk, as well as T cell function as measured by antigen specific cytokine secretion (13, 14). However, this work was performed on post-transplant samples only without any evaluation of pre-transplant immune phenotype that would allow for determination of differences occurring after transplantation as well as pre-transplant immune phenotype that might predict infection risk.

Here we sought to evaluate both pre- and post-transplant phenotype with a focus on markers of experience and senescence to develop a broader understanding of immune dysfunction and infection risk in kidney transplantation, including evaluation of the impact of patient age. Developing a better understanding of the immunologic effect of transplant and impact on infection risk is a necessary step towards development of individualized immunosuppression for transplant recipients.

## 2 Methods

### 2.1 Patient cohort

Patients were enrolled in a local IRB-approved observational study (UCLA IRB #11-001387). Subjects had blood drawn pre-transplant and then at regular post-transplant clinic visits followed by isolation and storage of PBMC as previously described (13, 14). The patient cohort was comprised of sequentially enrolled patients from the years 2019 to 2021 who had sufficient peripheral blood mononuclear cells (PBMC) available for analysis. Sequential enrollment was employed to reduce potential selection bias. Patient immunosuppression regimens and infection prophylaxis were as previously described, but to summarize briefly, patients received tacrolimus, mycophenolate, and prednisone immunosuppression with protocol-based monitoring of tacrolimus levels. Induction immunosuppression with either antithymocyte globulin (ATG) or basiliximab was selected based on donor quality and recipient history of sensitization. Antibiotic prophylaxis consisted of trimethoprim sulfamethoxazole and either valganciclovir for high risk (donor CMV seropositive, recipient seronegative [D+/R-] or recipient seropositive [R+] receiving ATG) or acyclovir plus preemptive monitoring for intermediate risk patients (R+ not receiving ATG) (14).

Chart review was performed to identify episodes of invasive infection using Infectious Diseases Society of America definitions for bacterial, fungal, and viral infections, including CMV. Patients were described in the “infection” group if they developed infection in the first year post transplantation, but “no infection” if they did not develop infection during this first year. Surveillance was performed every 3 months after transplantation for the first year.

### 2.2 Flow cytometry

Frozen PBMC were thawed, stained with fluorochrome-conjugated monoclonal antibodies, and fixed in Fluorofix buffer (Biolegend) using standard procedures and analyzed on a BD LSRFortessa. Protein targets and corresponding antibodies used in the CD4+ and CD8+ T cell panels are presented in **Supplementary Figure 1**. Raw FCS files were imported into R and analyzed as described below, using R packages ConsensusClusterPlus, flowCore, and flowWorkspace. A spillover matrix, defined using single-stain FCS files, was used to compensate for spillover between channels. Dead cells and doublets were removed, and raw MFI values were arcsinh transformed with a cofactor parameter of 150. CD3^+^CD4^+^ T cell and CD3^+^CD8^+^ T cell subsets were identified in an unsupervised manner using the FlowSOM algorithm, which initially defined 100 clusters using a Self-Organizing Map. For each of the two subsets, these clusters were combined into 40 meta-clusters by hierarchical clustering. For visualization, each subset was subsampled to 15,000 cells with equal representation per sample and per patient group. To ensure the subsampled cells per patient reflected the cluster distribution of their complete dataset, we generated 100 random subsamples per patient and chose the set which most closely matched the cluster distribution of the complete dataset. Normalized expression of 16 markers per panel for the subsampled cells was reduced to two dimensions, using t-distributed stochastic neighbor embedding (t-SNE), with perplexity values of 150 and 200 for CD4^+^ T cells and CD8^+^ T cells, respectively. Based upon manual inspection of the 40 meta-clusters per panel and their expression of canonical T-cell lineage markers, we defined naïve or memory-status of each meta-cluster and merged phenotypically similar meta-clusters. The final analysis included 28 CD4^+^ and 26 CD8^+^ T cell clusters.

### 2.3 Statistical analysis

R was utilized to perform statistical analysis (R Core Team 2021). Principal component analysis (PCA) analysis was performed on CD4 and CD8 T cells separately. Variable loadings for each T cell subset can be found in **Supplementary Data File 1** in Supplementary Data Sheet 1. Association between pre- versus post-transplantation or infection was performed using logistic regression. The association of freedom from infection, in the first year post-transplant, and cell types was assessed by Cox proportional-hazards models. For these models, patients were dichotomized per cell type around the percentage that maximized sensitivity and specificity in a receiver operating characteristic analysis predicting infection based on Youden’s index. A p-value of 0.05 or less was considered statistically significant. We used the STROBE cohort checklist when writing our report (15).

## 3 Results

### 3.1 Demographics and clinical outcomes

We evaluated 91 patients who had samples available for analysis. The median age of the cohort was 53, and patients were predominantly male with 51.0% of Hispanic ethnicity and 44.7% non-Hispanic white, 13.5% were Black and 9.4% Asian, similar to the overall kidney transplant population at our center (**Table 1**). The cause of kidney dysfunction was diabetes mellitus for 27.0% of patients and polycystic disease caused 12.4% of kidney disease, while 46.0% of patients had other diagnoses including chronic glomerulonephritis, obstructive uropathy, and unknown cause of disease. Most patients were on dialysis prior to transplantation, and 85.4% of patients underwent deceased donor transplant. 65.6% of patients received ATG induction. The majority of patients were receiving tacrolimus and mycophenolate mofetil (MMF) plus prednisone for maintenance immunosuppression.

**Table 1:** Cohort demographics.

Demographic characteristic	N=91
Median age (range)	53 (22-75)
Female sex	35.2%
Race/ethnicity:	
White	44.8%
Black	13.5%
Hispanic	51.0%
Median PRA	1.0%
Diagnosis:	
Diabetes	27.0%
Hypertension	9.0%
Polycystic kidneys	12.4%
IgA nephropathy	5.6%
Other	46.0%
Dialysis pre-transplant	94.4%
Retransplant	13.5%
Deceased donor	85.4%
Median KDPI	63.0
CMV antibody positive	69.2%
EBV antibody positive	93.3%

The rate of biopsy-proven rejection was 9.1% in the first year after transplantation (**Table 2**). The rate of infection in the first year was 52.8%, and included pneumonia, upper respiratory infection, pyelonephritis and urinary tract infection, gastroenteritis, cellulitis, and systemic infections including bacteremia. Causes of infection included bacteria (*E*. *coli*, *Klebsiella pneumoniae*, *Enterobacter*, *Enterococcus*, *Pseudomonas*, and *Clostridium difficile*), viruses (CMV, VZV, HSV, RSV, and SARS-CoV-2), and fungi (*Aspergillus* and *Candida*). Two patients died during the first year after transplantation.

**Table 2:** Clinical outcomes in the study cohort.

Clinical outcomes	N=91
ATG induction	65.6%
Immunosuppression:	
Tacrolimus and MMF	70.8%
Tacrolimus, no MMF	16.9%
Belatacept	11.2%
Other	1.1%
Acute rejection	9.1%
Infection	52.8%

### 3.2 T cell phenotypes impacted by immunosuppression

**Figure 1:**
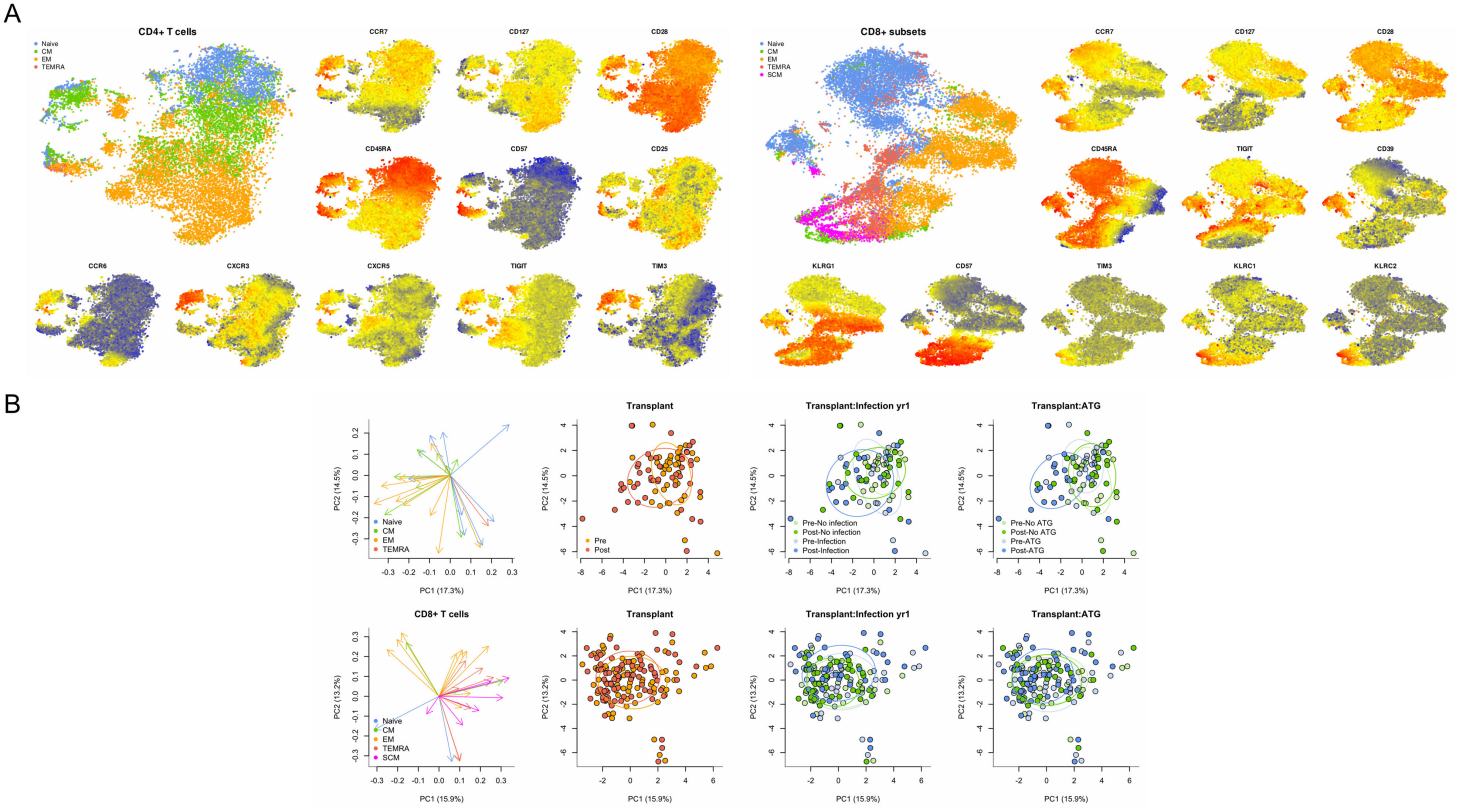
Comprehensive analysis of T cell phenotypes identified by flow cytometry. **(A)** TSNE analysis of CD4 and CD8 T cells by maturation subtype and selected markers including CCR7, CD127, and CD28 as indicated, with higher levels of expression indicated by red, intermediate by yellow, and lower levels indicated by blue. **(B)** Principal component (PC) analysis of CD4 and CD8 T cells mapped to specific maturation subtypes and demonstrated ability to discriminate patient samples collected before and after transplantation (red and orange dots) as well as patients who developed or did not develop infection (blue and green dots). Combination of transplant and infection status demonstrated additional discrimination ability.

The FlowSOM algorithm was paired with t-SNE analysis to identify clusters of phenotypically distinct subtypes within the CD4 and CD8 T cell populations (**Figure 1A**). Principal component analysis (PCA) was performed to evaluate the global association of T cell immune phenotypes with transplantation and infection (**Figure 1B**). For CD4, the first two principal components (PC) captured ~30% of the total variance. Central memory (CM) and effector memory (EM) CD4 T cell subsets generally were correlated on these PC, while naïve CD4 T cell subsets were more varied. This demonstrated ability to separate patient samples collected pre- versus post-transplantation for CD4 T cells, and discrimination by development of infection after transplantation and receipt of ATG induction therapy. Both CD4 and CD8 T cells were predominantly marked by decreased frequency of naïve cells and increased frequency of CM and EM post-transplant. For CD8, the first two PC similarly captured ~30% of the total variance. In contrast to CD4 T cells, differences in global CD8 T cell profiles did not reliably discriminate pre- vs post-transplant, development of infection, or receipt of ATG.

**Figure 2:**
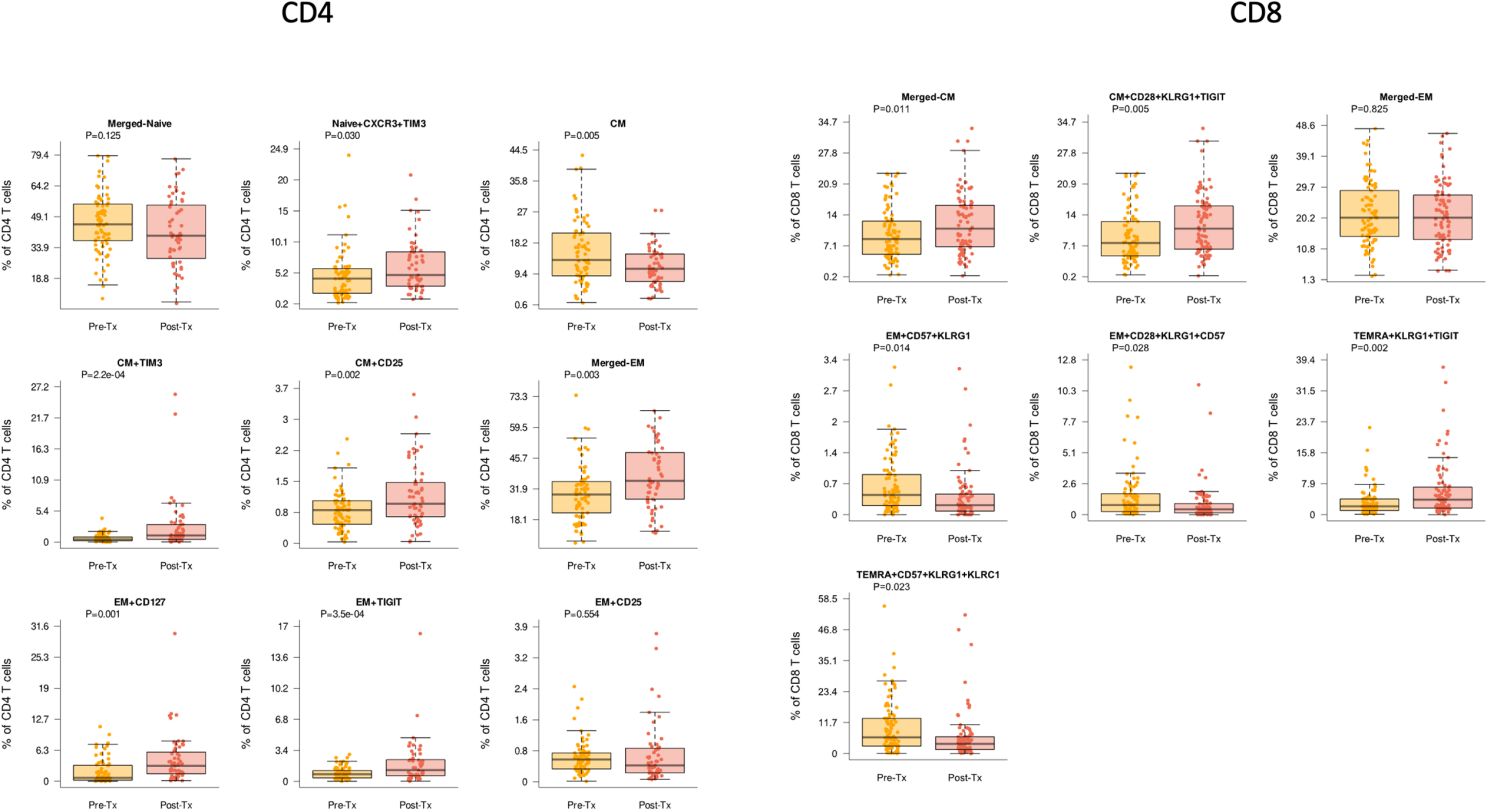
CD4 and CD8 T cells demonstrated differences pre- (yellow) compared with post-transplant (red) (Tx) across multiple cell subtypes. Bar and whiskers plot demonstrates median and IQR for frequency of T cells for each subtype. Y axes start at 0 for each graph. P values indicated as shown.

Evaluation of T cell maturation subtypes demonstrated several significant changes associated with transplantation and immunosuppression start. We observed a trend towards a decrease in naïve CD4 T cells after transplantation of 39.7% compared with pre-transplant frequency of 45.3% (p=0.125). For the naïve CXCR3+ TIM3+ CD4 subtype, a significant increase in frequency was observed (p=0.03), even with correction for patient age (**Figure 2**). Total frequency of central memory (CM) CD4 T cells were also decreased (p=0.005). However, specific CM CD4 T cells populations were noted to increase post-transplant, both TIM3+ CM (p<0.001) and CD25+ CM (p=0.002). The total CD4 effector memory (EM) population was also increased post-transplantation (p=0.003) (**Figure 2**). EM CD4 populations with markers for memory or senescence were also observed to increase after transplantation namely CD127+ (IL-7 receptor) (p=0.001), TIGIT+ (p<0.001), or CD127+TIGIT+ EM CD4 T cells (p<0.001). These differences also remained significant when corrected for patient age.

For CD8 T cell subtypes, we noted that stem cell memory (SCM) subtypes, defined as CCR7+ CD45RA+ cells expressing markers of activation and/or differentiation plus senescence markers were noted to decrease after transplantation. This included CD8 SCM CD57 KLRG1+KLRC2+ T cells (p=0.037) and CD8 SCM CD39+ TIGIT+ CD57+ KLRG1+ T cells (p=0.049) (**Figure 2**). Similarly, while the total population of CD8+ CM T cells did not change significantly after transplantation, the senescent subpopulations with upregulation of inhibitory receptors namely CD8+ CM CD28+ KLRG1+ TIGIT+ were noted to increase (p=0.005). Similarly, while the total population of CD8+ EM T cells did not change significantly after transplantation, senescent subpopulations did change significantly with transplantation, including CD8 EM CD57+KLRG1+ (p=0.014), EM CD28+KLRG1+CD57+ (p=0.028), and EM CD28+KLRG1+TIGIT+ (p=0.030), all of which retained significance with age correction (**Figure 2**). Overall CD8+ terminal effector memory re-expressing CD45RA (TEMRA) cells were not significantly changed, but senescent subpopulations were again significantly impacted, including increased TEMRA KLRG1+TIGIT+ (p=0.002) and decreased CD57+KRLG1+KLRC1+ (p=0.023).

### 3.3 T cell phenotypes impacted by ATG induction

**Figure 3:**
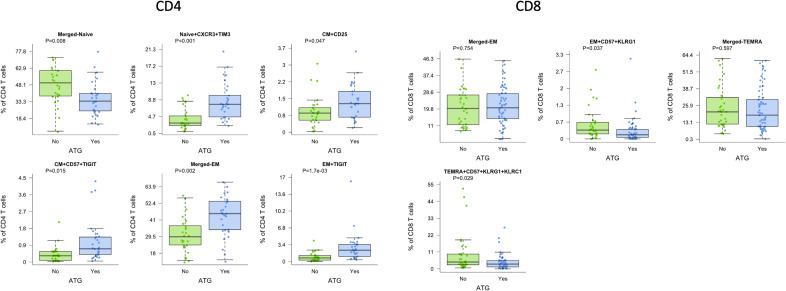
CD4 and CD8 T cells demonstrated differences by induction with ATG (green) compared with basiliximab (Simulect) (blue) across multiple cell subtypes. Bar and whiskers plot demonstrates median and IQR for frequency of T cells for each subtype. Y axes start at 0 for each graph. P values indicated as shown.

Lymphocyte depleting induction with ATG also demonstrated significant impact on T cell maturation subtypes compared with non-depleting induction with basiliximab (Simulect). We observed a decreased frequency of naïve CD4 T cells of 33.7% after ATG compared with 50.0% with basiliximab (p=0.008), which retained significance after correction for patient age (**Figure 3**). In contrast, we observed increased frequency of naïve CXCR3+TIM3 and CM CD57+TIGIT+ CD4 subtypes in patients receiving ATG compared with basiliximab (p=0.001 and p=0.015, respectively). CD4 EM T cells were also increased in patients receiving ATG, both for overall EM and TIGIT+ EM (both p=0.002).

For CD8 T cell subtypes, we noted significant differences in EM and TEMRA subtypes. Patients receiving ATG demonstrated significantly lower frequencies of activated CD8 EM CD57+ KLRG1+ T cells as well as lower frequencies of CD8 TEMRA CD57+ KLRG1+/KLRC1 positive T cells (p=0.037 and p=0.029, respectively), again demonstrating differential impact on senescent cells (**Figure 3**).

### 3.4 T cell phenotypes predictive of infection

**Figure 4:**
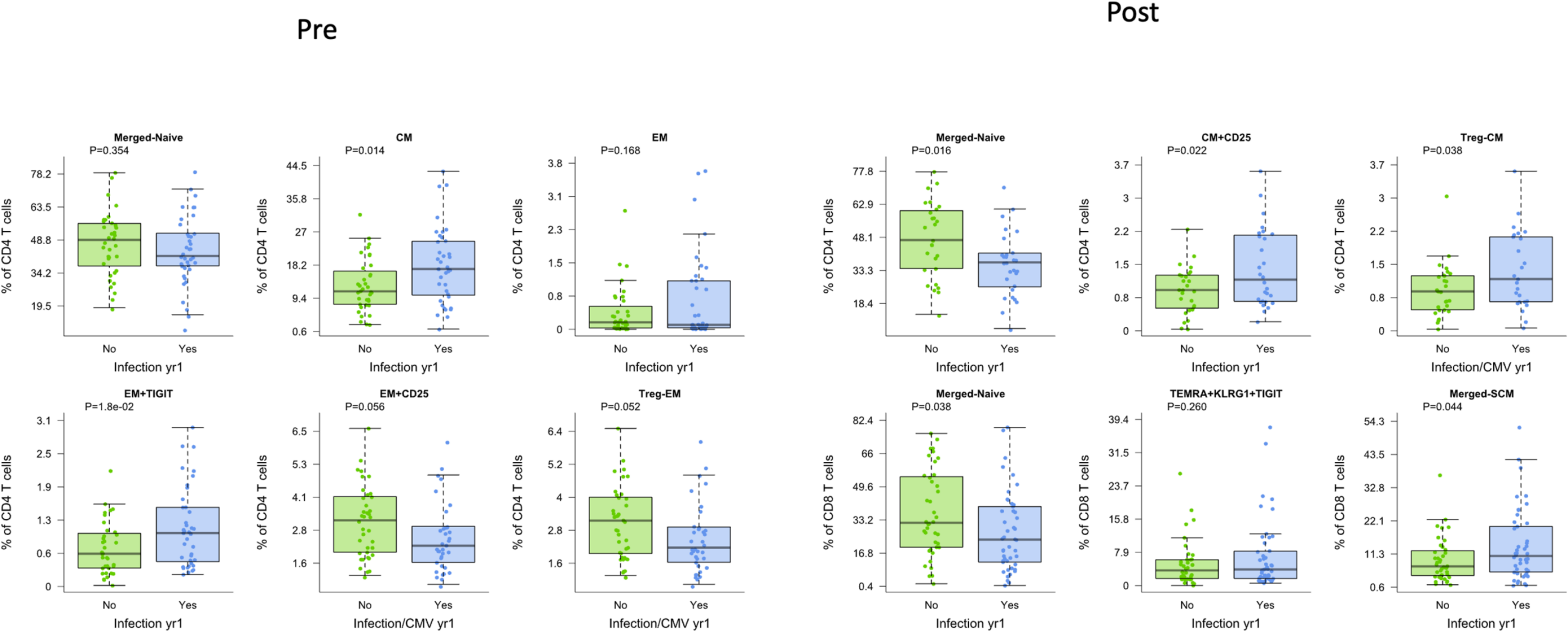
CD4 and CD8 T cells demonstrated differences in patients who were free from infection (green) or who developed infection post-transplant (blue), whether measured before (Pre) or after (Post) transplantation. Bar and whiskers plot demonstrates median and IQR for frequency of T cells for each subtype. Y axes start at 0 for each graph. P values indicated as shown.

Given the known association between T cell dysfunction and development of infection, we determined association with development of infection after transplantation. We observed that increased frequencies of CD4 CM and CD4 EM TIGIT+ T cells pre-transplant were associated with infection (p=0.014 and p=0.018, respectively), even with age correction (**Figure 4**, Pre). For the CD4 EM subtype expressing CD25, decreased frequency prior to transplant was associated with infection (p=0.056), although this observation only reached statistical significance with correction for patient age (p=0.019) (**Figure 4**, Pre). Given this association with CD25+ CD4 T cells, we examined the association with T regulatory cells (T regs, CD25+CD127-) measured prior to transplantation: We found a significant association with increased frequency of EM T regs and protection from infection (p=0.052 and p=0.017 with age correction). Increased frequency of EM TIGIT+ CD4 T cells demonstrated a trend towards association with infection (p=0.18). In contrast to the findings for CD4, CD8 subtypes measured prior to transplantation were not associated with post-transplant infection (**Supplementary Figure 2**).

Measurement of T cell phenotypes after transplantation was also found to be associated with development of infection. Decreased frequency of naïve CD4 T cells was strongly associated with infection (p=0.027) (**Figure 4**, Post). Increased frequency of total or CD25+ CD4+ CM T cells, in contrast, was associated with development of infection (p=0.033 and p=0.032, respectively). Analysis of Tregs also showed a significant association, with increased CD4 CM T regs post-transplantation associated with infection (p=0.038). Decreased frequency of naïve CD8 T cells was also associated with increased incidence of infection (p=0.029). Neither overall nor senescent subtypes of CD8+ TEMRA were associated with development of infection. Analysis of CD8 SCM subtype, however, demonstrated an association with development of infection (p=0.044) (**Figure 4**, Post).

We additionally visualized the relationship between frequency of these CD4 and CD8 T cell subsets and infection both pre- and post-transplant (**Supplementary Figure 2**). No populations were significantly associated with infection at both timepoints, suggesting that the mechanisms behind these relationships are impacted by transplant. However, frequencies of naïve CD4 and CD8 T cells pre-transplant did trend lower in those who experienced post-transplant infection.

For post-transplant sampling, due to sample availability, some patients were sampled before infection and others after infection. Therefore, we repeated the prior analysis factoring in the timing of samples around infection (**Supplementary Figure 3**). Findings were generally independent of whether the post-transplant sample was collected prior to or post infection, although the sample size per group was limited in this sub-analysis. The most significant difference was for CD4 CM expressing CD25, for which frequencies were only elevated before infection compared to uninfected controls.

### 3.5 Time-based analysis to predict infection

**Figure 5:**
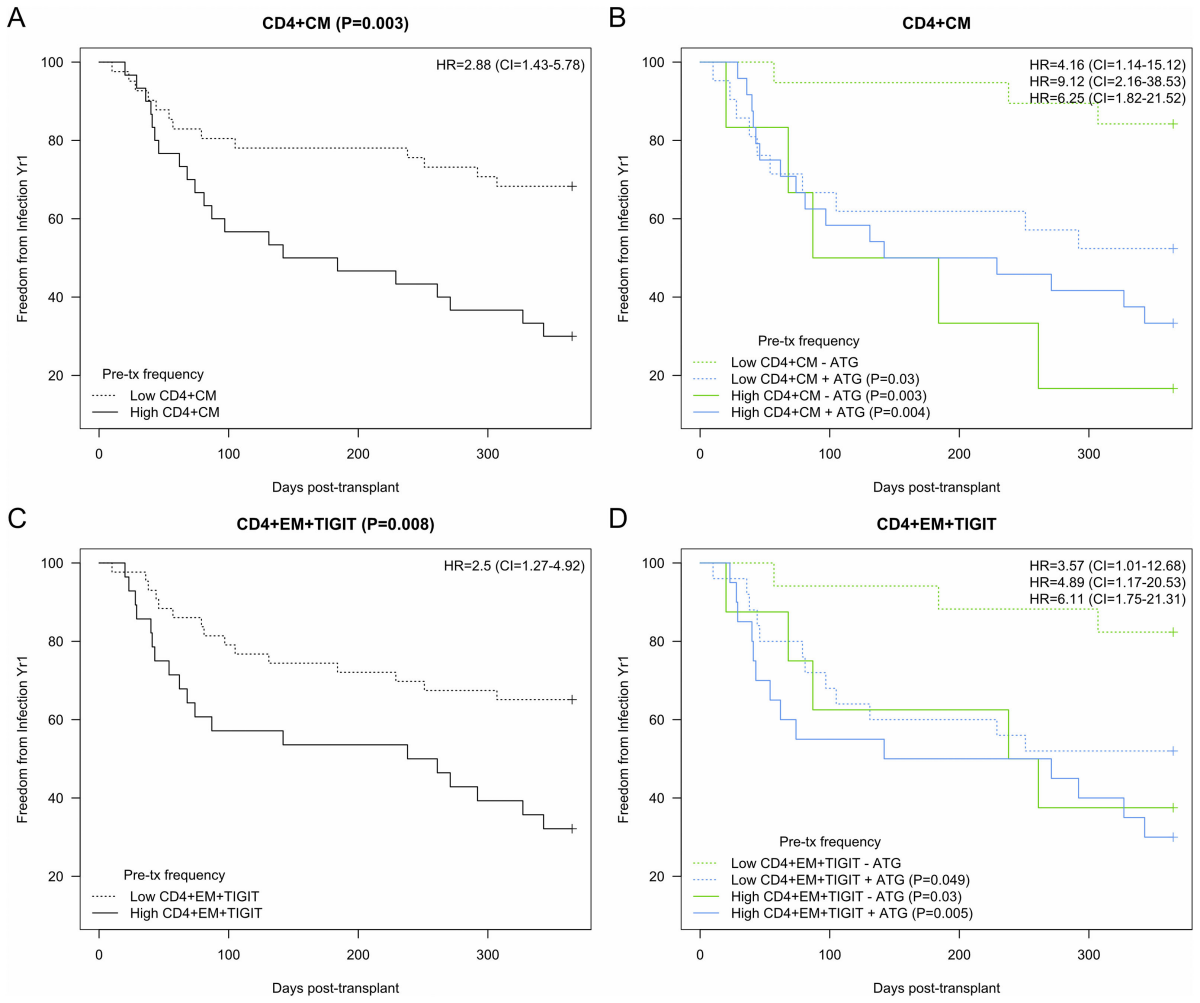
Kaplan-Meier analysis of CD4 T cell subtypes demonstrating association between subtype frequencies and development of infection after kidney transplantation. Hazard ratio (HR) and 95% confidence intervals (CI) as indicated. **(A)** CD4 Central memory (CM) with cutoff of 1.% designating ‘high’ demonstrated association with infection (HR 2.9, p=0.003). **(B)** High CD4 CM demonstrated association with (+) or without (-) ATG induction (HR 9.1 and 6.3, p=0.003 and 0.004). **(C)** CD4 Effector memory (EM) TIGIT+ with cutoff of 1.0% designating ‘high’ demonstrated significant association with infection (HR 2.5, p=0.008). **(D)** High EM TIGIT+ demonstrated association with (+) ATG induction (HR 6.1, p=0.005). Bold added for emphasis.

To extend the finding that specific T cell phenotypes were associated with infection, we determined cutoffs predictive of infection for several key CD4 T cell subtypes. Lower levels of CD4 CM T cells (<15.8%) were significantly associated with freedom from infection with an HR of 2.9 (95% CI 1.4-5.8) (p=0.003) (**Figure 5A**). When receipt of ATG induction was added to the model, it retained statistical significance, with patients with lower levels of CM T cells and the absence of ATG induction at the lowest risk for infection compared with the other three groups (p ≤ 0.03) (**Figure 5B**). Lower levels of CD4 EM TIGIT+ T cells were also predictive of freedom from infection with and HR of 2.5 (95% CI 1.3-4.9) (**Figure 5C**). Similarly to CD4 CM t cells, with the addition of ATG induction as a co-variate we found that the combination of lower level of EM TIGIT+ in the absence of ATG induction was the strongest predictor of freedom from infection (p ≤ 0.05) (**Figure 5D**).

## 4 Discussion

We found that T cell phenotypes are impacted by transplantation and lymphodepleting induction immunosuppression and are associated with risk of post-transplant infection when measured both before and after transplantation. Specifically, we uniquely found that senescent T cell subtypes expressing TIGIT or TIM3 were impacted by transplantation and that TIGIT+ EM CD4 T cells, in conjunction with induction type, could predict development of infection. Concurrently, naïve CD4 and CXCR3+ naïve T cells decreased after transplantation. CXCR3 expression may characterize naïve T cells responding to stimuli after lymphodepletion, as part of the process of homeostatic proliferation (16). CXCR3 expression may demonstrate a pre-activation phenotype in naïve T cells which may be upregulated after transplantation. Given that the ligands for CXCR3 are CXCL9 and CXCL10, the mechanism may be related to ability to migrate and interact with antigen presenting cells (17–19). The observation that pre-transplant immune phenotypes can predict infection suggests that patient-level differences in terms of varying degrees of senescence related to chronic kidney disease play an important role in predicting impact of immunosuppression and risk for post-transplant infection. These studies demonstrating association with specific T cell phenotypes predictive of infection suggest that the mechanism by which immunosuppression impacts vulnerability to infection is via upregulation of specific inhibitory surface molecules.

Interestingly, an increase in experienced CM CD4 T cells and CM CD4 T cells expressing CD127 or TIM-3, which were higher after transplantation and with ATG induction, was associated with increased risk of infection. CD127 (IL-7R) is associated with persistence of memory and homeostatic proliferation of CD4 memory cells (20). It has been suggested that TCR ligation may inhibit signaling through IL-7 and IL-7R (21). TIM-3 is a negative regulator that blocks IFN-g expression and is expressed in Th1 CD4+ T cells, as well as promoting development of exhausted CD8+ T cells (22). Therefore, TIM-3 may be a marker of exhaustion in CD4 T cells, possibly triggered by cytokine secretion in the setting of inflammation post-transplantation. Another potential explanation is that TIM-3 represents an activated cell type, suggesting an association between antigen-experienced T cells and development of infection. Notably, in the context of chronic viral infection, virus-specific T cells can express TIM-3, but their ability to produce antiviral cytokines is diminished (10). Future work should focus on functionally profiling these T cells to delineate the balance between activation and exhaustion.

TIGIT is a member of the CD28 family and while it can be upregulated upon activation, it has been shown to possess inhibitory functions (10). CD226 (DNAM-1) engagement is associated with T cell activation; TIGIT blocks this process by competing for ligand, leading to T cell inhibition (23). The observation that TIGIT expression is associated with immunosuppression start, lymphodepleting induction, and infection therefore suggests that upregulation of this marker may be an important pathway underlying infection vulnerability due to immunosuppression. Cells expressing two or more inhibitor receptors such as TIGIT and KLRG1 have sometimes been termed ‘exhausted-like’ or ‘partially exhausted’ T cells that are impaired in antigen response despite absence of PD-1 expression and have shown to be predictive of response to immunologic therapy for diabetes (24). In our study, these potentially exhausted T cell populations increased in frequency after transplantation. Engagement of alloantigens with CD4 T cell TCR from the transplanted allograft or autoantigens uncovered by ischemia reperfusion injury after transplantation is a possible mechanism through which TIGIT becomes upregulated prior to exposure to infection-related antigens. As described above for TIM-3, future functional profiling would help define whether these memory cells remain activated and proliferative, or whether TIGIT expression has driven them towards a more regulatory function.

We also observed that the frequency of CD4 CD25+ (IL-2R) T cells increased after transplantation. This may reflect the fact that IL-2 is known to be a trigger for homeostatic proliferation, so that memory T cells expressing this receptor may be able to better proliferate after transplantation (25). We also found that increased frequency of these CD25+CD4 T cells and CD4 Tregs (CD25+CD127-) with a CM T cells were protective against infection. This may represent the fact that a subset of these memory cells are specific for viral or bacterial antigens and proliferate in response to exposure to infectious antigens. Increased frequency of Tregs is associated with immune aging and control of infection, especially for viral infections, through the mechanism of modulating antigen presentation and interfering with costimulatory molecule expression (26, 27).

A recently identified T cell subtype found to be significantly associated with infection was CD8 stem cell-like memory cells. This T cell population is not well understood but have been defined by some researchers as a subtype of exhausted T cells expressed early in both acute resolving and chronic infection (28). We additionally noted a trend towards association with infection for stem cell-like memory T cells expressing markers of exhaustion (CD39 and CD244) and senescence (CD57) (29).

**Figure 6:**
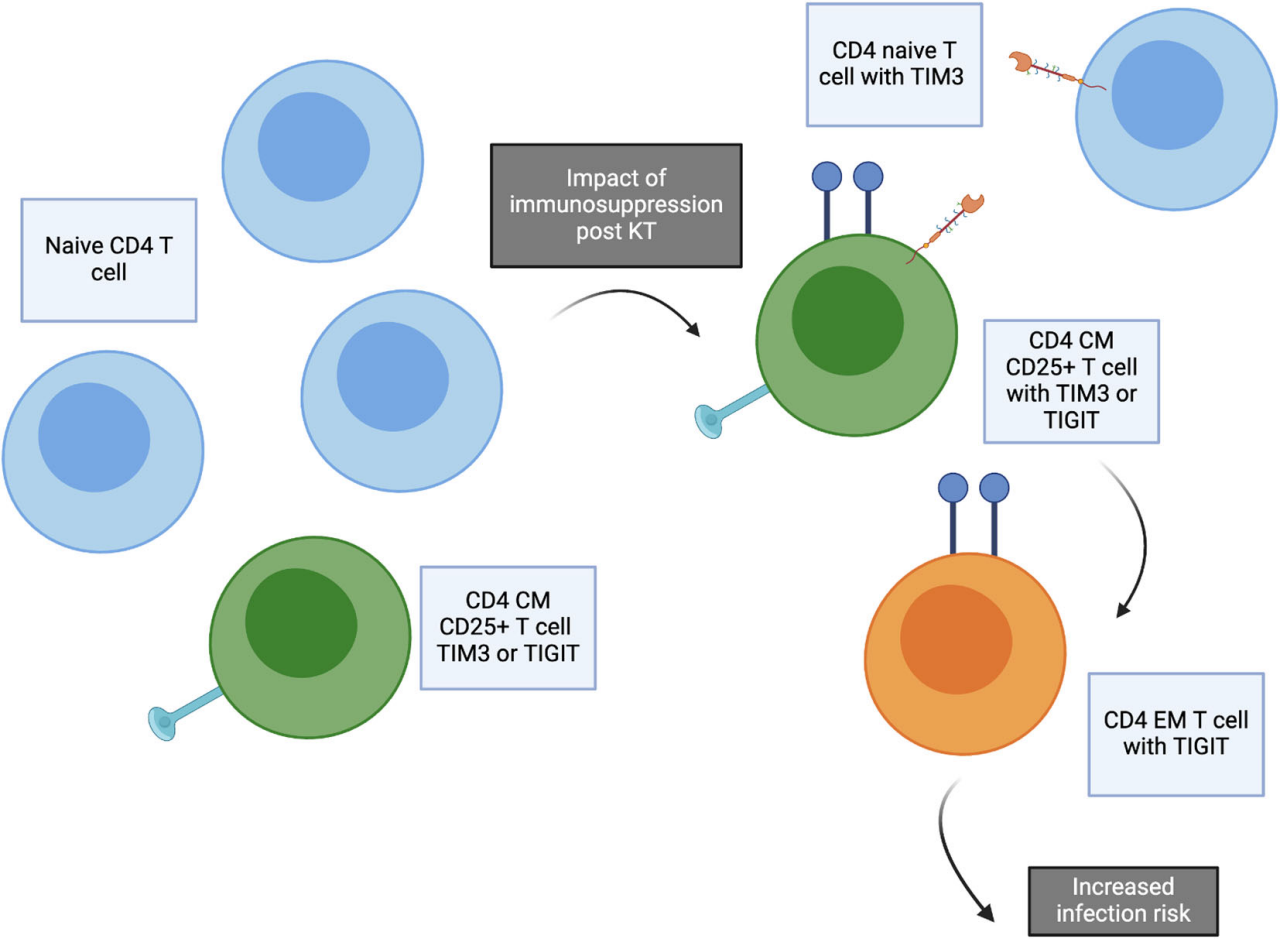
Concept figure demonstrating impact of transplantation and immunosuppression start. The frequency of naïve CD4 T cells decreases after transplantation, especially for patients receiving ATG induction. Post kidney transplantation, increased frequency of CD4 naïve T cells expressing TIM3, CD4 central memory (CM) CD25+ T cells expressing TIM3 or TIGIT, and CD4 effector memory (EM) T cells expressing TIGIT are significantly increased. These T cell subtypes are associated with increased risk of infection after transplantation.

Analyses of immune phenotypes impacted by transplantation and those associated with infection demonstrates several key cell types associated both with immunosuppression start and vulnerability to infection: These subtypes were defined as CM CD4 T cells, with or without CD25 expression, and TIGIT+ EM CD4 T cells. Interestingly, there appears to be a time-based difference in the impact of these phenotypes on infection risk, with CD4 T cell frequency demonstrating a difference quite early post KTx while (~50–100 days) while the impact of CD4+EM+TIGIT appears to be later (~200–250 days) (**Figure 5**). The CD4 CM and EM TIGIT cell types were also predictive of freedom from infection in a time-based analysis, especially with incorporation of induction immunosuppression with ATG (**Figure 6**). This analysis demonstrates that transplantation including immunosuppression start and use of lymphodepleting induction impacts the frequency of these cell types and alters risk for infection after kidney transplantation. Future studies will validate this connection and attempt to determine whether changes in induction or maintenance immune suppression impacts frequency of CD4 T cell subtypes defined as conferring infection risk, as well as whether these subtypes might impact rejection risk.

The PCA analysis integrating all aspects of immune phenotype for both CD4 and CD8 T cells further demonstrated the global impact of transplantation and the association with infection across T cell phenotypes. This analysis demonstrates that several CM CD4 T cell subtypes clustering in PC2 appear to have a coordinated impact, including CD4 CM, CD4 CM TIGIT, and CD4 CM CXCR3, in contrast with the subtypes CM CD57 TIGIT, EM TIGIT, EM CD25, and EM CD127 TIM3 in PC3 (**Figure 1**). These analyses suggest interconnection across multiple T cell phenotypes impacted by transplantation and predictive of development of infection. Evaluation of markers found to be in common between association with both transplant impact and infection confirmed the key role of CD4 CM, CM 25+ and EM TIGIT T cells in both impact of transplantation and prediction of infection (**Figure 6**).

Limitations to this work include the number of patients assessed as well as the fact that only a single post-transplant time point was evaluated. A larger cohort size would permit more detailed statistical analyses including ability to further evaluate the relationship between immune phenotype pre- and post-infection. The impact of heterogeneity of patients studied is lessened by the implementation as a single center study with uniform protocols for managing immunosuppression and monitoring for infection. While TIM-3 and TIGIT have both been linked to exhaustion and senescence in T cells previously, future work performing functional profiling of these T cell subsets should be performed to confirm their functional capacities, specifically whether they retain proliferative capacity or are more regulatory. The assessment of PBMC by targeted flow cytometry panels somewhat limits the ability to understand the full spectrum of T cell subtypes within the populations studied; future evaluations can utilize higher-resolution approaches in combination with gene expression assessment to better characterize cell populations given the heterogeneity of the CD4 and CD8 T cell compartments. Additional future studies can assess memory T cells by functional ability to control viral infection, antigen specificity and clonotypic expansion within the kidney and blood compartment. Future studies can also evaluate epigenetic changes underlying T cell phenotype changes.

We found in this cohort of kidney transplant recipients that transplantation had a significant and a specific impact on certain T cell subtypes independent of patient age. In addition, specific subtypes were significantly associated with development of infection after transplantation. Further investigations can determine whether this approach can be applied to individualize immunosuppression and risk stratify patients when T cell populations are evaluated before or after transplantation.

## Data Availability

The raw data supporting the conclusions of this article will be made available by the authors, without undue reservation.
