# Supplementary material for: TIM3 and TIGIT-expressing CD4 T cells are impacted by kidney transplantation and associated with risk of infection
Source: Front Immunol. 2025 May 22;16:1550154. doi: 10.3389/fimmu.2025.1550154 (PMC12137242; doi:10.3389/fimmu.2025.1550154)

**Supplementary File:**

TIM3 and TIGIT-expressing CD4 T Cells Are Impacted by Kidney Transplantation and Associated with Risk of Infection

**Supplementary Figure 1:** Markers used for immune phenotyping panels.


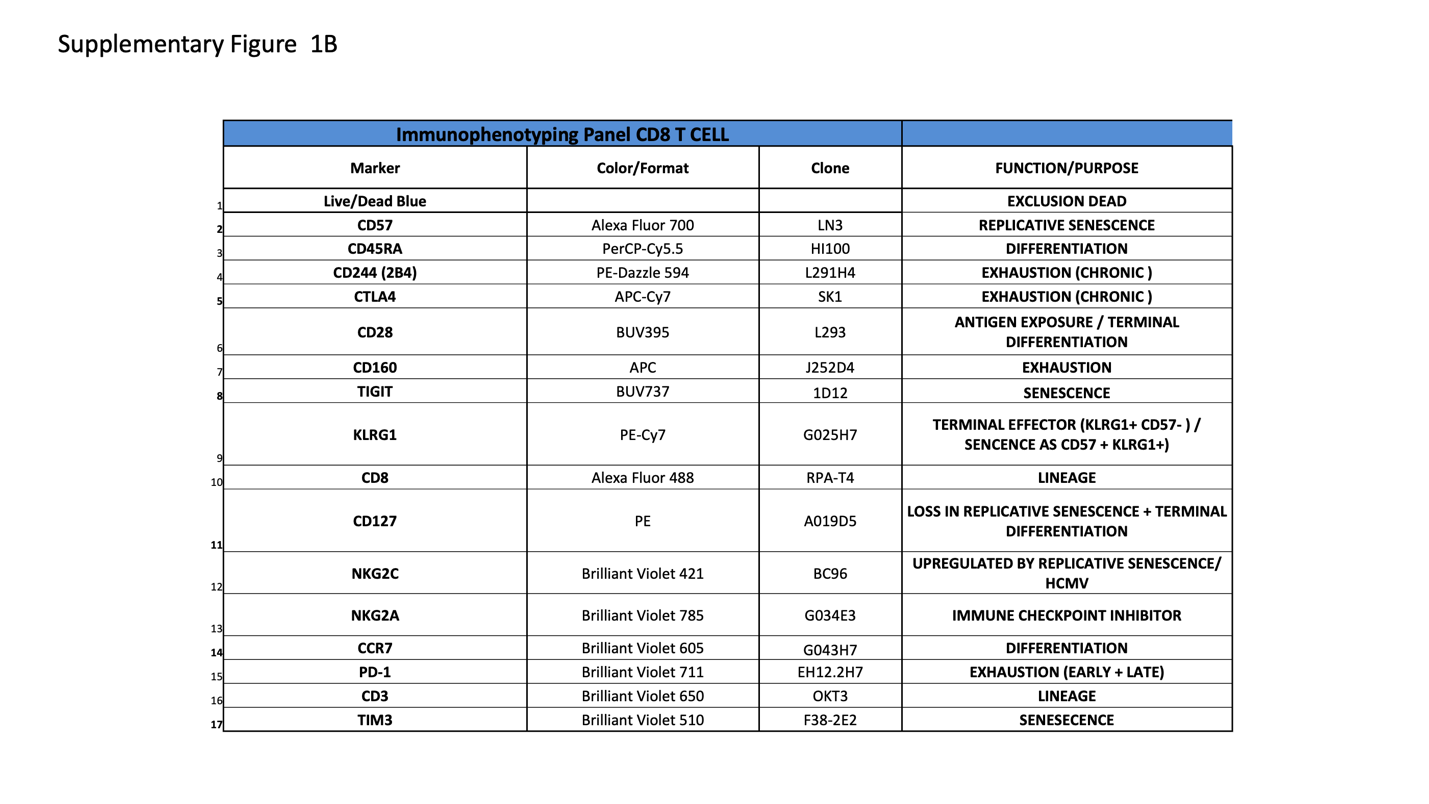

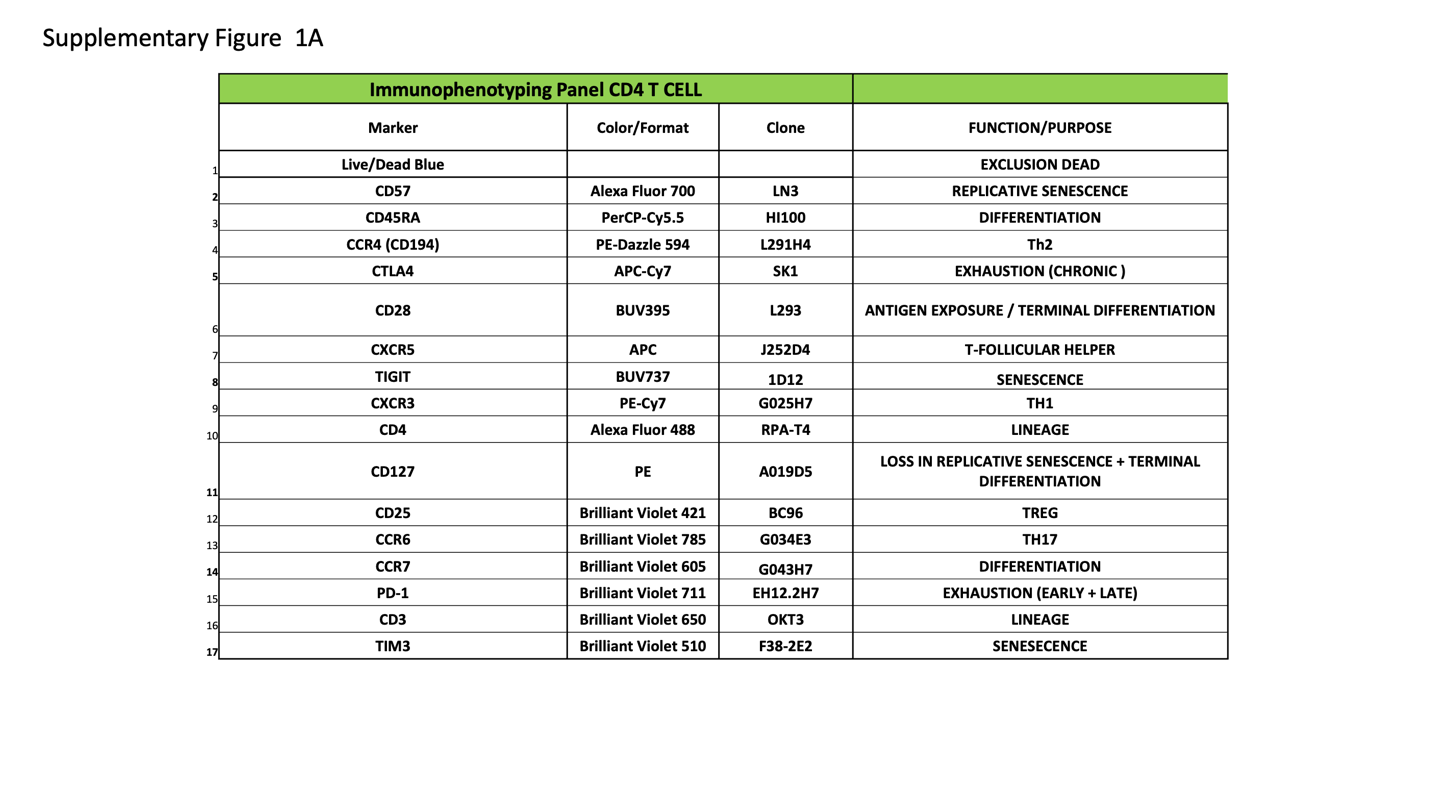


**Supplementary Data File 1.** PCA loadings for CD4 and CD8 T cell analysis presented in Figure 1.

| Population | PC1 | PC2 |
| --- | --- | --- |
| CD4+Naive+TIGIT | -0.0355336 | 0.20374531 |
| CD4+Naive | -0.0973975 | 0.18860796 |
| CD4+Naive.1 | 0.28525172 | 0.2396073 |
| CD4+Naive+CXCR3+TIM3 | -0.2011517 | -0.0171745 |
| CD4+CM+TIM3 | -0.1647519 | -0.0586021 |
| CD4+EM+CD127+TIM3 | -0.2255189 | -0.142054 |
| CD4+EM+CD127+CD25 | -0.0955168 | -0.1897656 |
| CD4+Naive+CD25 | -0.1515543 | 0.11465713 |
| CD4+CM | -0.0193488 | 0.0456818 |
| CD4+EM | 0.14582586 | -0.3408381 |
| CD4+EM+CD57 | -0.0567445 | -0.3679973 |
| CD4+CM+CD25 | -0.2700951 | -0.0071622 |
| CD4+EM+CD25 | -0.2618507 | -0.1237318 |
| CD4+EM+CD127 | -0.1766495 | -0.0193575 |
| CD4+CM.1 | 0.03470884 | 0.07490054 |
| CD4+TEMRA+CD57 | 0.18446073 | -0.2382441 |
| CD4+Naive+CD57+CD25 | 0.21204703 | -0.2181699 |
| CD4+CM+CD57+TIGIT | -0.3167361 | -0.1864115 |
| CD4+CM+CD57+CD25 | 0.05360576 | -0.2918884 |
| CD4+CM+CXCR5 | -0.0639177 | 0.10364261 |
| CD4+EM+TIGIT | -0.0927613 | 0.15095056 |
| CD4+Naive+CD57+CD25.1 | 0.15609892 | -0.3299943 |
| CD4+Naive+CD57+CD25+CXCR3+TIM3 | 0.06889388 | -0.2830067 |
| CD4+Naive+CD57+CXCR3+TIM3 | 0.05674153 | -0.1875562 |
| CD4+CM+TIGIT | -0.1601745 | 0.12185882 |
| CD4+EM+TIGIT.1 | -0.3674345 | -0.1334109 |
| CD4+EM+CD25.1 | -0.332487 | -0.0506199 |
| CD4+EM+TIGI+CXCR5 | -0.2451946 | -0.0141872 |

| Population | PC1 | PC2 |
| --- | --- | --- |
| CD8+EM+CD28+CD127+TIGIT | -0.2035868 | 0.28615147 |
| CD8+EM+CD28+CD127+TIGIT+KLRG1 | -0.1819897 | 0.31908089 |
| CD8+EM+CD28+KLRG1+TIGIT | 0.11282108 | 0.23359578 |
| CD8+EM+CD28+KLRG1+TIGIT+CD57 | 0.13451277 | 0.22729142 |
| CD8+EM+CD28+KLRG1+CD57 | 0.15135987 | 0.01469807 |
| CD8+SCM+CD57+KLRG1+KLRC2 | 0.3391766 | 0.09225723 |
| CD8+TEMRA+CD57KLRG1+KLRC1 | 0.15342908 | -0.061181 |
| CD8+CM+CD28+KLRG1+TIGIT | -0.1588306 | 0.27008266 |
| CD8+EM+CD57+TIGIT | 0.08304646 | 0.21424903 |
| CD8+EM+CD57+KLRG1+TIGIT | 0.23941432 | 0.25262366 |
| CD8+EM+CD57+KLRG1 | 0.110042 | -0.0578326 |
| CD8+TEMRA+CD57 | 0.09538016 | 0.04053802 |
| CD8+EM+CD28+CD127 | -0.2505343 | 0.23488566 |
| CD8+TEMRA+CD57+KLRG1+TIGIT | 0.26367867 | 0.09512924 |
| CD8+TEMRA+KLRG1+TIGIT | 0.21076251 | 0.14223636 |
| CD8+TEMRA+KLRG1+TIGIT.1 | 0.12891561 | 0.17876936 |
| CD8+TEMRA+CD57+KLRG1 | 0.09999724 | -0.3213896 |
| CD8+TEMRA+CD57+KLRG1+TIGIT+KLRC1 | 0.10028919 | -0.3275323 |
| CD8+Naive | -0.3131163 | -0.1682571 |
| CD8+SCM+KLRC1+KLRC2+TIGIT+CD57+KLRG1 | 0.30807322 | -0.0076388 |
| CD8+Naive+TIGIT | 0.06217994 | -0.328965 |
| CD8+SCM+CD39+TIGIT+CD57+KLRG1 | 0.11403022 | -0.1457724 |
| CD8+SCM+CD39+CD57 | 0.19291132 | -0.0718861 |
| CD8+SCM+CD39+CD57+CD244 | -0.0597104 | -0.0894424 |
| CD8+CM+CD57+KLRG1+KLRC2 | 0.3091877 | 0.07867785 |
| CD8+SCM+KLRC1+KLRC2+TIGIT+CD57 | 0.25075974 | 0.07601686 |

**Supplementary Figure** 2. CD4 and CD8 T cell phenotypes in patients who were free from infection (green) or who developed infection post-transplant (blue), measured before (Pre, left panels) or after (Post, right panels) transplantation. Bar and whiskers plot demonstrates median and IQR for frequency of T cells for each subtype. Y axes start at 0 for each graph. P values indicated as shown.


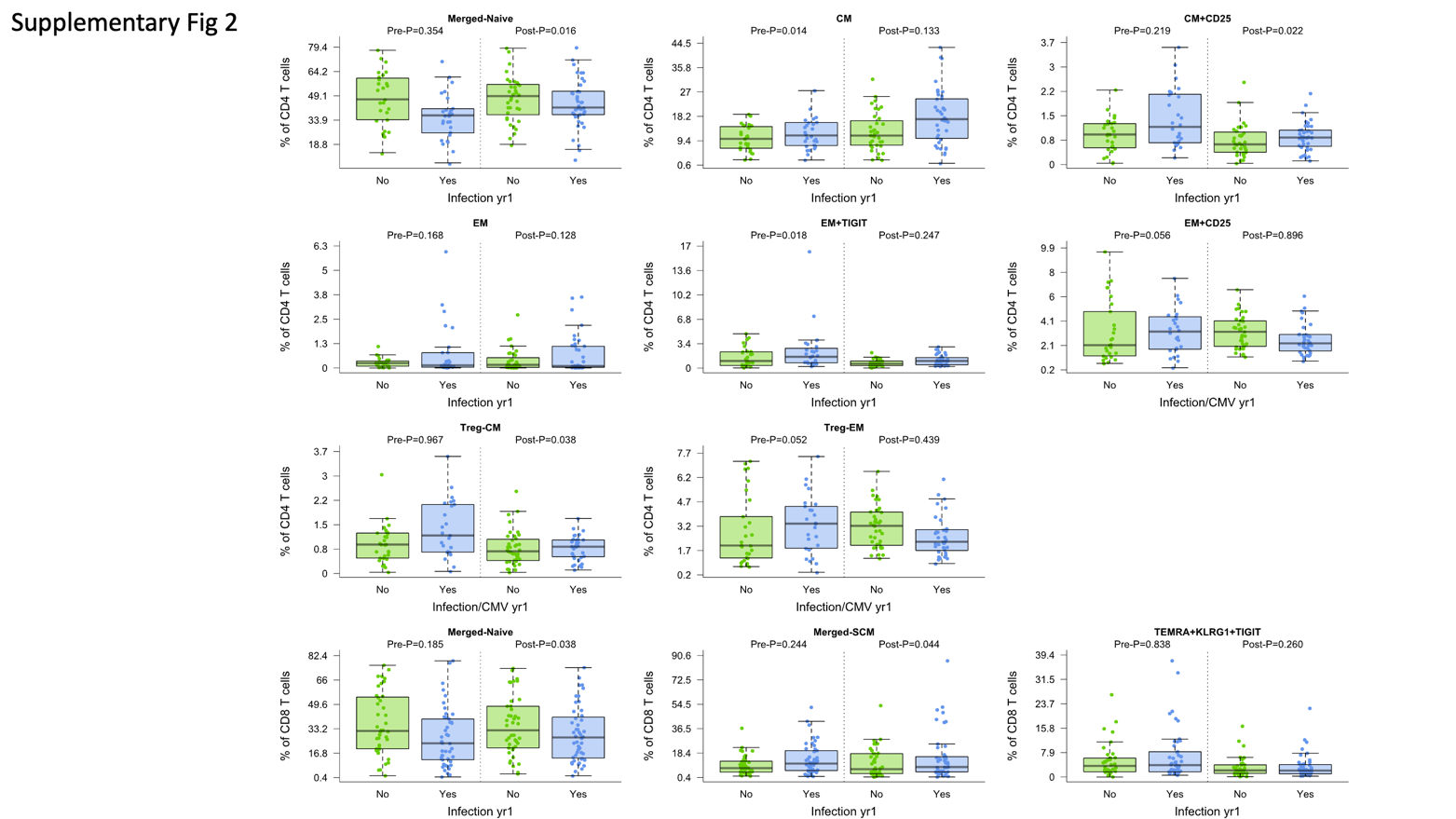
 **Supplementary Figure 3.** CD4 and CD8 T cells demonstrated differences in patients who were free from infection (green) or who developed infection post-transplant (blue), whether measured before (light blue) or after (dark blue) infection. Bar and whiskers plot demonstrates median and IQR for frequency of T cells for each subtype. Y axes start at 0 for each graph. P values indicated as shown.


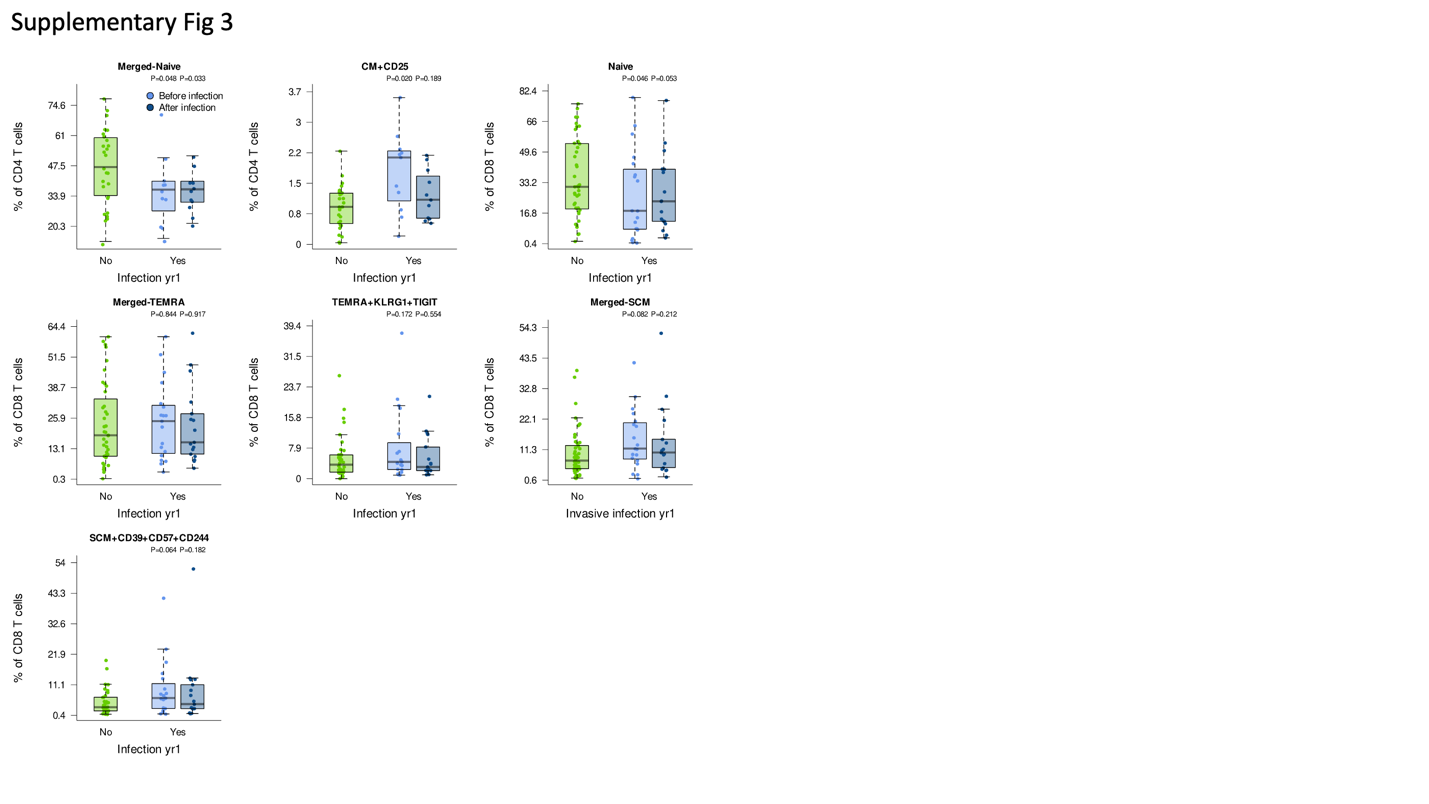

Supplement: Supplementary file 1 [file DataSheet1.docx]
